# Adénocarcinome primitif de la vessie: à propos de 6 cas

**DOI:** 10.11604/pamj.2020.36.61.20176

**Published:** 2020-06-03

**Authors:** Youssef Kadouri, Farouk Hachem, Jihad Lakssir, Hachem Sayegh, Lounis Benslimane, Yassine Nouini

**Affiliations:** 1 Université Mohamed V, Faculté de Médecine et de Pharmacie de Rabat Maroc, Hôpital Ibn Sina, Service d´Urologie A, Rabat, Maroc

**Keywords:** Mots clés: Vessie, tumeur rare, adénocarcinome, Bladder, rare tumor, adenocarcinoma

## Abstract

L´adénocarcinome primitif de la vessie est une variété rare de tumeurs vésicales qui représente moins de 2% des cancers de la vessie. Il semble atteindre préférentiellement le sexe masculin avec un sexe ratio de 3/1 et un âge moyen de survenu entre 60 ans et 70 ans. Sa présentation clinique est non spécifique et dominée par l´hématurie. La résection endoscopique de la vessie avec un examen anathomopathologique permet de poser le diagnostic. Le traitement de l´adénocarcinome primitif de la vessie reste sujet à de nombreuses controverses du fait de la rareté des cas rapportés dans la littérature. Cependant, le traitement de choix semble être une cystectomie totale avec curage ganglionnaire étendu. Nous rapportons une série de 6 cas d´adénocarcinome qui ont été traités et suivis au sein de notre formation. Notre analyse est basée sur l´évaluation des caractéristiques épidémiologiques, cliniques, anatomo-pathologiques, et thérapeutiques de l´adénocarcinome de la vessie, ainsi que sur l´étude des aspects évolutifs et des facteurs pronostiques.

## Abstract (en)

Primitive adenocarcinoma of the bladder is a rare form of bladder tumor accounting for less than 2% of bladder cancers. It mostly affects male sex, with a sex ratio of 3/1 and an average age of onset between 60 years and 70 years. Clinical manifestation is non-specific and dominated by haematuria. Endoscopic resection of the bladder with anatomo-pathological examination allows the diagnosis. Treatment of primitive adenocarcinoma of the bladder is controversial due to the rarity of cases reported in the literature. However, the treatment of choice seems to be radical cystectomy with extended lymphadenectomy. We report a series of 6 cases of adenocarcinoma treated and followed-up in our hospital. Our analysis is based on the evaluation of the epidemiological, clinical, pathological and therapeutic features of adenocarcinoma of the bladder as well as on the study of the evolutionary features and prognostic factors.

## Introduction

La tumeur de la vessie représente le 2^e^ cancer le plus fréquent du tractus uro-génital après le cancer de la prostate [1], son incidence est de plus de 300 000 cas par an dans le monde ce qui représente 5 à 8% de tous les cancers [2]. Le carcinome urothélial transitionnel représente 90-95% des tumeurs malignes de vessie [3]. Les tumeurs non urothéliales de la vessie, aussi bien bénignes que malignes, forment des entités beaucoup plus rares et représentent moins de 5% de tous les néoplasmes vésicaux, parmi lesquelles on trouve l'adénocarcinome (ADK) primitif vésical qui représente le 3^e^ cancer par ordre de fréquence après le carcinome urothéliale et le carcinome épidermoide [4]. C´est une entité rare qui représente 0,5 à 2% de toutes les malignités de la vessie [5,6]. Il survient le plus souvent chez l´homme (sexe ratio de 3/1), entre la 5^e^ et la 6^e^ décade [7,8]. Malgré leur faible incidence, les urologues doivent y penser devant toute tumeur de vessie, en particulier celle avec présentation clinique inhabituelle.

## Méthodes

Notre travail est une étude rétrospective des dossiers médicaux, étalée sur une période de 10 ans concernant six cas d´adénocarcinome vésical, diagnostiqués, traités et suivis au Service d´Urologie A du CHU de Rabat. Notre analyse est basée sur l´évaluation des caractéristiques épidémiologiques, cliniques, anatomo-pathologiques, et thérapeutiques de l´adénocarcinome de la vessie, ainsi que sur l´étude des aspects évolutifs et des facteurs pronostiques. Les données recueillies pour la réalisation de ce travail proviennent des dossiers des patients dans les archives du service et des comptes rendus opératoires.

## Résultats

**Figure 1:**
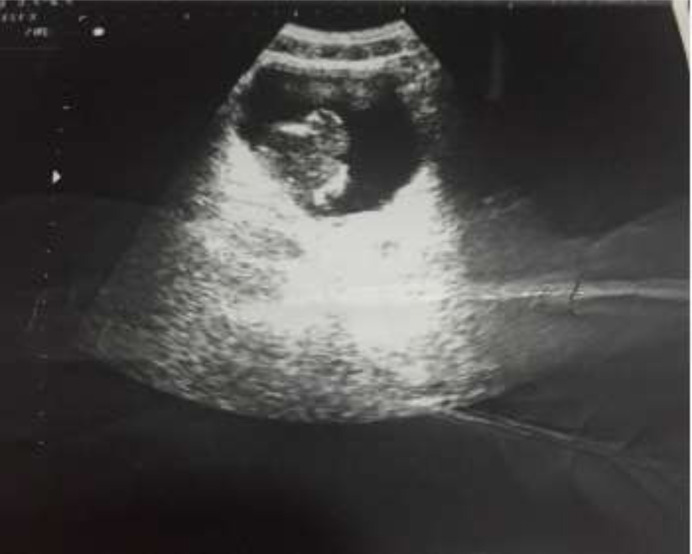
cliché d'échographie montrant un épaississement tumoral bourgeonnant à l'intérieur de la vessie

**Figure 2:**
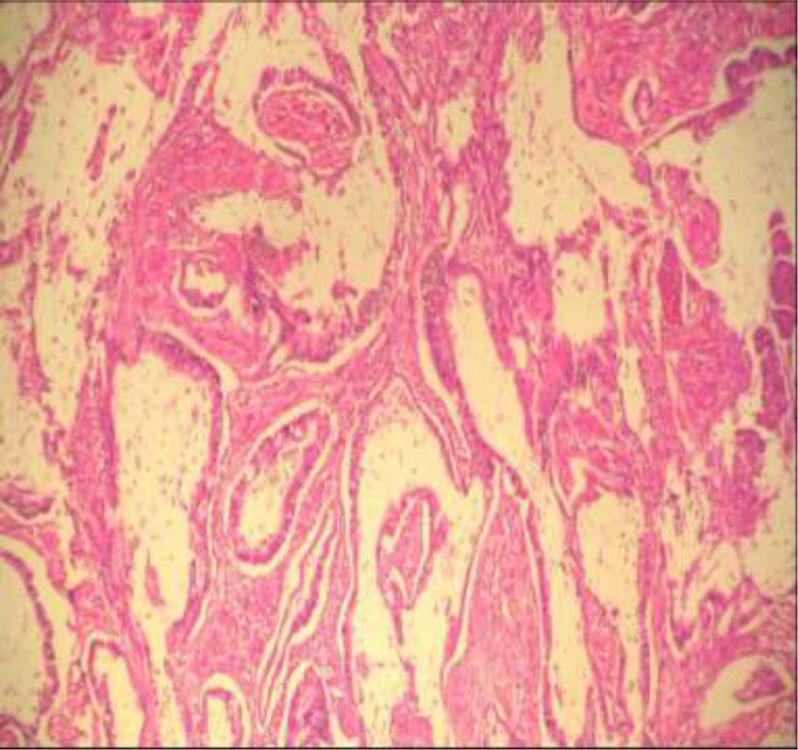
cellules tumorales disposées en tubes irréguliers au sein d'un stroma inflammatoire comprenant de vastes plages de mucus (HE x 100)

**Figure 3:**
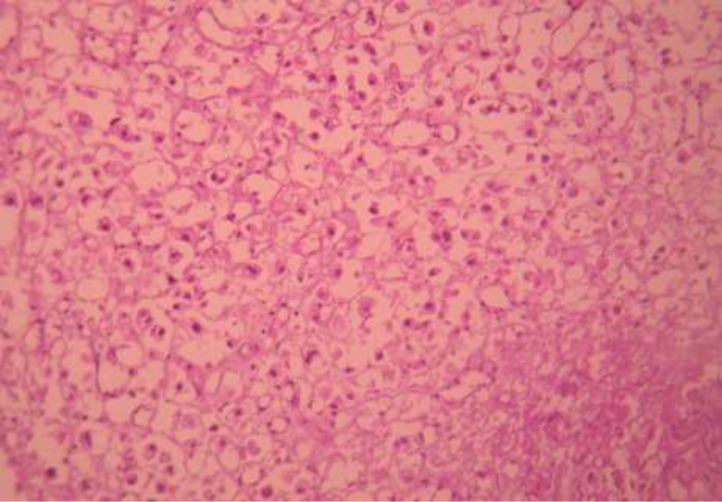
prolifération carcinomateuse faite de cellules de grande taille a cytoplasme vacuolaire réalisant un aspect de cellules en bague à chaton

**Figure 4:**
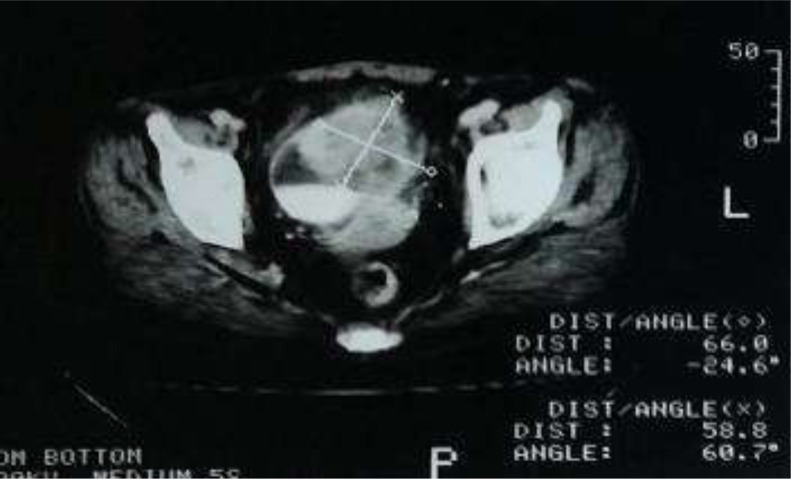
cliché de TDM montrant un processus tumoral vésical de 66x58 mm intéressant le dôme et la paroi antérolatérale gauche avec infiltration de la graisse péri-vésicale

Il s´agit de 5 hommes et une femme, l´âge moyen était de 58,3 ans avec des extrêmes allant de 46 ans à 74 ans. Un antécédent de tabagisme chronique a été trouvé chez deux des 6 patients (33,3%); avec une consommation moyenne de 7 paquets-année, les autres facteurs de risque à savoir la bilharziose urinaire, les infections urinaires à répétition et la vessie neurologique n´ont pas été retrouvés dans notre série. Le maitre symptôme était une hématurie totale avec des caillots, retrouvée chez tous les patients, associé à des signes irritatifs du bas appareil urinaire dans 66,66% des cas (4 malades), des signes obstructifs dans 50% des cas (3 malades), une altération de l´état général (AEG) avec anorexie et amaigrissement non chiffré dans 50% des cas (3 malades) et une hémorragie digestive basse dans 33% des cas (2 patients). L´examen clinique a été anormal dans 50% des cas: une masse hypogastrique chez 2 patients, une cicatrice de chirurgie antérieure chez 1 patient et une infiltration de la paroi vaginale antérieure chez la femme. Sur le plan paraclinique: une anémie a été retrouvée chez 3 patients, soit 50%, avec un taux d´hémoglobine allant de 7 g/dl à 12,3 g/dl, secondaire à la maladie néoplasique, ainsi qu´à la spoliation sanguine par hématurie. Cette anémie a imposé une transfusion sanguine dans 2 cas soit chez 33% des patients anémiques. Une insuffisance rénale a été retrouvée chez un patient qui s´est amélioré après réhydratation. Sur le plan radiologique: tous nos patients ont été examinés par une échographie réno-vésicale qui avait permis d´explorer la morphologie vésicale et de suspecter le diagnostic d´une tumeur de vessie en objectivant une masse tissulaire pariétale (Figure 1) chez 5 patients (non concluante chez un patient: vessie pleine de caillots). La résection trans-urétrale de la vessie (RTUV), a été faite chez tous nos patients au service, elle a été complète chez 2 patients et incomplète chez 4 car la tumeur a été jugée incontrôlable endoscopiquement. Elle a été unique chez tous les patients. L´examen anatomo-pathologique des copeaux de résection a permis de poser le diagnostic de certitude d´adénocarcinome vésical chez tous nos malades: 4 cas d´adénocarcinome mucineux (soit 66,66% des cas) (Figure 2), un cas d'adénocarcinome à cellules en bague à chaton (Figure 3), et un cas d´adénocarcinome à cellules claires. Le scanner thoraco-abdomino-pelvien dans le cadre du bilan d´extension a été réalisé chez tous nos patients et a révélé: une tumeur localement avancée associée à des métastases pulmonaires et ganglionnaires chez 3 malades (Figure 4), soit dans 50% des cas, avec envahissement de la jonction recto-sigmoïdienne chez une malade. La cystectomie totale associée à un curage ganglionnaire ilio-obturateur bilatéral a été réalisée chez 3 patients, suivie d´un geste de dérivation urinaire par enterocystoplastie chez un patient et un bricker chez 2 patients. Une chimiothérapie palliative à base de 5 fluoro uracile a été proposée chez les 3 autres patients, cependant réalisée que chez 2, le 3^e^ patient avait refusé le traitement. Les suites opératoires immédiates étaient compliquées d´un choc hémorragique chez un patient nécessitant une hospitalisation en réanimation et d´un accident vasculaire cérébral ischémique chez un deuxième patient à J2 post-opératoire. L´évolution à long terme était favorable avec un recul de 2 à 3 ans chez 2 patients soit 33,33% des cas et défavorable chez 2 autres patients (rechute métastatique à 6 mois chez le premier avec des métastases hépatiques, surrénaliennes droites et rétro-péritonéales, décès chez le deuxième par détresse respiratoire aigüe). Deux patients ont été perdus de vue.

## Discussion

L´adénocarcinome primitif de la vessie est une variété rare de tumeurs vésicales et représente moins de 2% des cancers de la vessie. Il s´agit de la troisième variété histologique la plus fréquente des cancers de la vessie après le carcinome urothélial et le carcinome épidermoïde [4]. Il prédomine chez l´homme avec un âge moyen de survenu entre 60 ans et 70 ans [7,8]. L´adénocarcinome vésical primitif est classé en adénocarcinome ouraquien et non ouraquien. La différenciation entre ces deux types repose essentiellement sur des critères cliniques et morphologiques, le phénotype immuno-histochimique étant le plus souvent peu informatif et superposable [9]. L´adénocarcinome ouraquien se développe souvent au niveau de la paroi postérieur de la vessie où du dôme vésical, à partir des reliquats de l´ouraque, son épicentre se situe au niveau du muscle vésical et tend à s´étendre vers l´espace de Retzius formant une masse sus pubienne. Il présente souvent des calcifications en pointillés qui peuvent être identifiées sur l´uroscanner [4]. Tandis que la forme non ouraquienne se développe souvent dans la base de la vessie à partir d´une métaplasie de l´urothélium [8].

L´étiopathogénie de cette tumeur reste hypothétique et très controversée. Son développement au sein d´un épithélium normalement dépourvu de toute structure glandulaire a fait avancer de nombreuses théories, la théorie métaplasique semble unir la majorité des auteurs, et tire son originalité du pouvoir métaplasique du revêtement urothélial de la vessie [10], qui a lieu sous l´effet de facteurs irritatifs mécaniques ou chimiques [7,8,11]. Le support de ce mécanisme vient des cas survenant chez des patients avec métaplasie intestinale diffuse de la muqueuse vésicale associée à une obstruction, cystocéle, vessie neurologique, exstrophie vésicale, entérocystoplastie ou à une irritation chronique (infections et inflammations chroniques) [12,13]. En cas de transplantation rénale, l´incidence de ce type de tumeur augmente, et l´âge au moment de leur survenue diminue, ce qui s´explique par les traitements immunosuppresseurs utilisés [14]. Cependant, cette tumeur pourrait également se développer à partir des cellules épithéliales pluripotentes [15]. Sur le plan histologique, cette lésion est caractérisée par des lésions tumorales formant une structure glandulaire qui ressemble à l'adénocarcinome colique (Figure 2). Grignon a classé l´adénocarcinome primitif non ouraquiens de la vessie en 6 types histologiques [8]: ADK entérique, ADK mucineux (colloïde), ADK à cellules en bagues à chaton, ADK à cellules claires (mésonéphrique), ADK mixte et ADK indifférencié. Dans notre série, on a eu la chance d´avoir la plupart des types histologiques décrits: un patient présentait la variante à cellules en bagues à chaton, 4 patients présentaient l´adénocarcinome mucineux, un seul patient avait un ADK à cellules claires et aucun patient ne présentait un ADK de type entérique et indifférencié. La symptomatologie clinique de l´adénocarcinome n´est pas spécifique et diffère peu de celle du carcinome urothélial, dominée par l´hématurie macroscopique qui est retrouvée dans 90% des cas, il s´y associe fréquemment des signes d´irritation vésicale à type de pollakiurie, brûlures mictionnelles. La mucosurie, signalée dans environ 1/4 des cas, est très évocatrice d´une telle tumeur [16].

A la cystoscopie, l´adénocarcinome de la vessie se présente le plus souvent comme une lésion unique à la différence du carcinome urothélial qui a tendance à être multifocal et se localise le plus souvent au niveau du trigone et la paroi postérieure comme une lésion papillaire, solide ou ulcéreuse, mais toute autre localisation est possible [17]. La RTUV avec l´étude histologique permet de poser le diagnostic positif. L´analyse immunohistochimique des copeaux de résection par les marqueurs (cytokératine CK7 et CK 20 CDX2, et surtout β-caténine) est important pour différencier un adénocarcinome primitif de vessie et un adénocarcinome secondaire (le plus souvent, envahissement par une tumeur colo-rectale) [17]. Une expression positive de la protéine CDX-2 et de la cytokératine 20 (CK 20) associée à une expression négative de la cytokératine 7 (CK 7) est très évocatrice d´un adénocarcinome du tractus digestif. Wang a montré que la dysrégulation de la β-catenine permettant cette différentiation: son expression nucléaire est positive dans les tumeurs colo-rectales envahissant la vessie et négative dans les adénocarcinomes primitifs de la vessie [18]. Sur le plan paraclinique, la tomodensitométrie (TDM) thoraco-abdomino-pelvienne est indispensable pour évaluer l´extension tumorale locorégionale, le retentissement sur le haut appareil urinaire et déterminer l´extension métastatique. La cytologie urinaire et la recherche d´une mucosurie est positive dans 20% des cas. Malgré le nombre des modalités thérapeutiques utilisées dans les différentes publications, le traitement de l´adénocarcinome de la vessie reste sujet à de nombreuses controverses du fait de la rareté des cas rapportés dans la littérature d´où l´intérêt d´une approche multidisciplinaire dans des centres spécialisés. La cystectomie partielle a été proposée pour les tumeurs localisées sur la partie mobile de la vessie, mais les résultats étaient souvent très décevants [5,6,19]. Paradoxalement, Anderstrom a rapporté une survie à 5 ans de 54% chez 15 malades traités par cystectomie partielle. Mais, cet auteur signale, par ailleurs, une survie de 21% chez 7 malades traités par irradiation. C´est ainsi que la cystectomie totale, associée à un curage ganglionnaire étendu, demeure le traitement de référence avec une médiane de survie à 5 ans de 35% tous stades confondus [20]. L´expérience de la chimiothérapie systémique est très limitée dans la littérature. A partir des résultats obtenus pour les adénocarcinomes coliques, une chimiothérapie à base de 5-fluorouracile (5-FU) a été proposée dans beaucoup de publications avec des résultats différents [21,22,23]. Une chimiothérapie neo-adjuvante à base de sels de platine peut montrer son efficacité aussi selon certains auteurs en cas de tumeurs localement avancées, en permettant une réduction de la taille tumorale avant un éventuel traitement chirurgical [24]. Le pronostic de l´adénocarcinome primitif reste péjoratif: la survie à 5 ans, tous stades confondus, s´échelonne de 0 à 33% [25]. Les trois facteurs pronostic principaux sont: le stade, le grade et le degré d´envahissement ganglionnaire.

## Conclusion

L´adénocarcinome primitif de la vessie est une tumeur rare, de pronostic sombre, qui présente un défi diagnostique et thérapeutique et doit bénéficier d´efforts concertés de plusieurs disciplines médicales pour établir une stratégie thérapeutique efficace et améliorer le pronostic.

### Etat des connaissances actuelles sur le sujet

- L´adénocarcinome de la vessie est une tumeur très rare;
- Sa prise en charge thérapeutique est mal codifiée vu la rareté des cas rapportés dans la littérature.

### Contribution de notre étude à la connaissance

- Il faut penser au diagnostic de l´adénocarcinome de la vessie même en absence de facteurs de risque connus de cette pathologie (aucun facteur de risque n´a été trouvé dans notre série);
- Confirme la place de la cystectomie totale avec un curage ganglionnaire tenu comme traitement de référence de cette tumeur rare;
- L´entérocystoplastie garde sa place comme mode de dérivation urinaire chez les patients en bon état général, pris en charge rapidement.
